# Artificial Intelligence and our journal, "Einstein São Paulo"

**DOI:** 10.31744/einstein_journal/2026ED2690

**Published:** 2026-07-22

**Affiliations:** 1 Hospital Israelita Albert Einstein São Paulo SP Brazil Hospital Israelita Albert Einstein, São Paulo, SP, Brazil.

Our institution, *Hospital Israelita Albert Einstein*, has been using Artificial Intelligence (AI) long before the advent of large language models. For example, our emergency department has used Isabel, an AI-based diagnostic program, for several years. Artificial Intelligence holds significant promise, particularly in freeing healthcare professionals from routine and repetitive tasks. A systematic review evaluated the use of large language models in healthcare applications,^(1)^ while a scoping review^(2)^ and an umbrella review on the impact of ChatGPT in healthcare^(3)^ further explored this topic. Additionally, medical education may benefit from the use of tools such as ChatGPT.^(4)^

How should we approach the use of AI in manuscripts submitted to our journal? Several publications have addressed the role of large language models in scientific writing.^(5)^ In our view, the use of such tools to improve the quality of English is acceptable, particularly because many of our authors are not fluent in written English. However, the use of AI must be clearly disclosed. We do not support the use of AI to write scientific reports, as this practice has several pitfalls. AI-generated text may inadvertently reproduce entire passages from previously published work, raising concerns about plagiarism. We employ systems to detect such issues, and methods are now available to quantify the use of large language models in scientific manuscripts.^(6)^

We also discourage the use of AI to conduct systematic or narrative reviews. Large language models tend to aggregate information from multiple databases without critical evaluation, increasing the risk of combining high-quality evidence with unreliable or low-quality data. Hallucinations, that is fabricated or incorrect information, are common. The appropriate approach to preparing a review remains unchanged: identifying relevant studies, reading them in full, not only abstracts, and carefully selecting those that meet rigorous standards. Although this may seem like a traditional approach, it remains the most reliable. AI-assisted reference generation may even lead to citations of non-existent articles; we have encountered such cases. The most effective way to organize references continues to be with the assistance of a trained librarian or through the use of reference management software. It is important to note that all references are checked.

Artificial Intelligence systems retrieve existing published knowledge but do not distinguish between articles from reputable peer-reviewed journals and those from predatory, pay-to-publish outlets. This lack of discrimination is a significant limitation of these tools.

In conclusion, AI will undoubtedly contribute to advances in many areas of medicine and health sciences. However, producing high-quality, original scientific work suitable for publication in this journal is unlikely to be one of them.

## Data Availability

Data are available to reviewers upon request.
